# Description and biology of two new species of Neotropical *Liriomyza* Mik (Diptera, Agromyzidae), mining leaves of *Bocconia* (Papaveraceae)

**DOI:** 10.3897/zookeys.369.6168

**Published:** 2014-01-13

**Authors:** Stéphanie Boucher, Kenji Nishida

**Affiliations:** 1 Department of Natural Resource Sciences, McGill University, Macdonald Campus, Ste-Anne-de-Bellevue, Quebec, H9X 3V9, Canada; 2 Escuela de Biología, Universidad de Costa Rica, 2060 San José, Costa Rica

**Keywords:** Biocontrol of weeds, *Bocconia arborea*, *Bocconia frutescens*, leaf miner, *Liriomyza mystica*, *Liriomyza prompta*, Hawai`i, Neotropical, parasitoid wasps, systematics, taxonomy, Tree poppy

## Abstract

*Liriomyza mystica* Boucher & Nishida, **sp. n.**, and *Liriomyza prompta* Boucher & Nishida, **sp. n.** are described from Costa Rica. Both species were reared from leaves of *Bocconia frutescens* L. (Papaveraceae). The latter species was also reared from *B. arborea* S. Watson. Larvae of *L. mystica* mine primary veins of large, relatively old, mature leaves, and *L. prompta* mine blades of small to large, mature leaves. These represent the first record of agromyzids feeding on *Bocconia*. Biological information is also given and illustrated.

## Introduction

*Liriomyza* Mik is the second largest genus of agromyzid flies (after *Phytomyza* Lioy) with approximately 390 described species worldwide. Due to its high diversity, small size and mostly uniform external characters, identification at the species level is sometimes difficult for this genus. Host plant association is often an important tool for species identification especially for host-specific species. Host plants are known for 177 species (45%) of *Liriomyza* (Benavent-Corai et al. 2005) with almost all species feeding as leaf miners, except a few species that develop in stems, flower buds or potato tubers (Parrella 1987). Most species are monophagous or oligophagous, although several of the most highly polyphagous agromyzid species belong to this genus. As many as 76 plant families have been recorded as hosts for *Liriomyza* species, which is the widest host range known among Agromyzidae (Spencer 1990). In the Neotropical region, *Liriomyza* is the dominant genus with 92 species described (*Liriomyza avicenniae* Martinez; *Liriomyza pectinimentula* Sasakawa and *Liriomyza pervensis* Zlobin added to species list in Martinez and Etienne 2002). Host plants are known for approximately one-third of these species (Benavent-Corai et al. 2005) with some considered to be of major economic importance due to the damage they cause to ornamental plants and cultivated crops (Spencer 1973) or for their highly polyphagous habits (Boucher 2010). Species of Papaveraceae have previously been recorded as host plants for six agromyzid species (Benavent-Corai et al. 2005): *Phytomyza horticola* Goureau (on *Eschscholzia* Cham., *Glaucium* Miller, *Papaver* L.) *Liriomyza strigata* (Meigen) (on *Glaucium*, *Meconopsis* Viguier and *Papaver*), *Calycomyza jucundacea* (Blanchard) (on *Papaver*), *Liriomyza huidobrensis* (Blanchard) (on *Papaver*), *Liriomyza xanthocera* (Czerny) (on *Papaver*), and *Phytomyza parvicella* (Coquillett) (on *Papaver*), but none were previously recorded from the genus *Bocconia*.

*Bocconia frutescens* L., commonly known as Tree poppy, Parrotweed, Plume poppy, Sea oxeye daisy or simply *Bocconia* is a large shrub to small tree native to tropical America occurring from Mexico to Argentina, and Bahamas (Wagner et al. 1999; Hammel et al. 2007). It is easily recognized by its relatively dry and soft trunk and deeply lobed leaves reaching 30 cm wide and 55 cm long or larger (90 cm) (Hammel et al. 2007, K. Nishida, pers. observation). The species was intentionally introduced as an ornamental plant in Hawai`i in the early 1900’s (Wester 1992), but has now been listed as a noxious weed by the State of Hawai`i (Benitez and Saulibio 2007). In Costa Rica, the species has been recorded between 100 and 3300 m on both Atlantic and Pacific slopes (INBio 1997–2006; Hammel et al. 2007), and is commonly found in middle to high elevation cloudforests, along road sides, river banks, open fields, and light gaps (K. Nishida, pers. observation). According to Hammel et al. (2007), a second species of *Bocconia*, *Bocconia arborea* S. Watson occurs in Costa Rica—it has the same appearance as *Bocconia frutescens* except for deeper lobed foliage with more whitish texture on the underside. It has a smaller distributional range, occurring from Mexico to Costa Rica, possibly to Panama at an elevation varying from 1100 to 1600 m.

Here we describe two new species of *Liriomyza* reared from *Bocconia* at various localities in Costa Rica.

## Materials and methods

**Figures 1–7.**
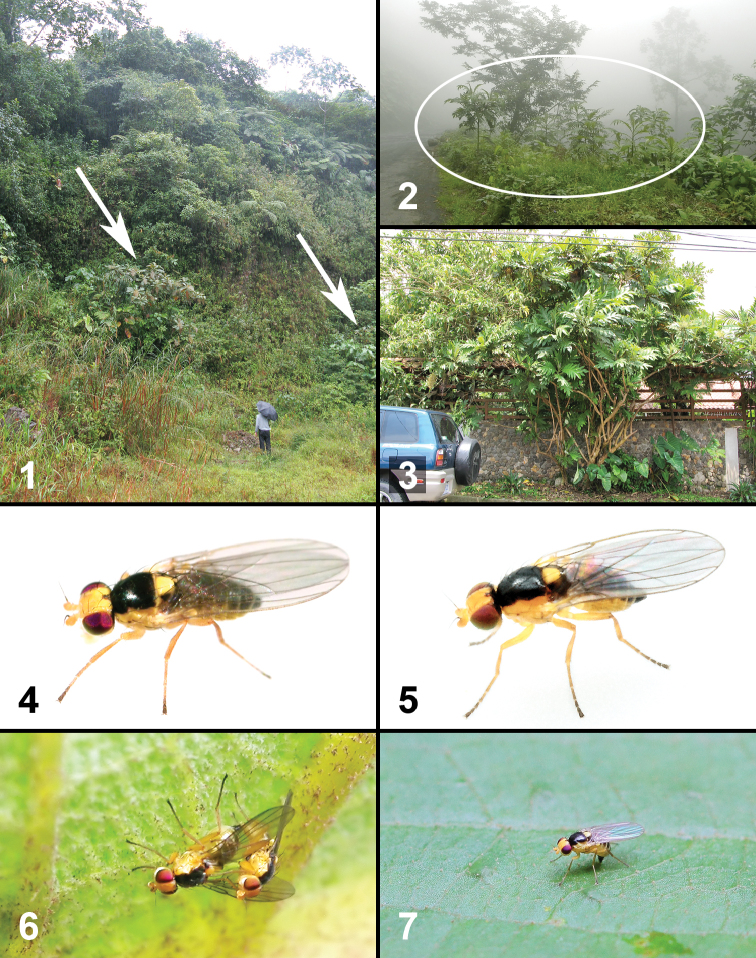
Life history of two new species of *Liriomyza*. **1–3** Habitats **1** Open area in a valley near Reserva Biológica Manuel Alberto Brenes in San Ramón (site 2). Arrows indicate *Bocconia frutescens* trees **2** *Bocconia frutescens* saplings (in circle) growing along the road after land slides caused by 2009 earthquake in Cinchona-Vara Blanca area (site 10) 3 Ornamental *Bocconia frutescens* tree (in middle) in urban area of San Isidro de Coronado (site 15) **4** *Liriomyza mystica* female (from site 5) **5** *Liriomyza prompta* female (from site 13) **6** Mating couple of *Liriomyza prompta* on the underside of *Bocconia frutescens* leaf at 7:00 am (30.v.2009, site 13) **7** *Liriomyza prompta* ovipositing on the upper side of *Bocconia frutescens* leaf blade at 3:00 pm (17.vi.2011, site 2).

Leaves of *Bocconia frutescens* and *Bocconia arborea* infested by the *Liriomyza* species have been collected at multiple localities in Costa Rica (Figs 1–3, Table 1). Most of the observation, collecting and rearing was conducted between April 2007 and July 2011. The leaves collected were taken back to a laboratory in San Isidro de Coronado (Table 1, site 15) for rearing. Approximate average temperature of the rearing chamber was 25 °C (day) and 18 °C (night). Infested parts of the leaf blade were separated from the primary vein in order to separate the two fly species, which are site-specific on the leaf. The leaf blade and the primary veins were placed either in large transparent plastic bags or translucent Tupperware for observation and rearing of the larvae, puparia and parasitoids. Most adults were obtained from rearing, except a few caught while mating on *Bocconia frutescens* at site 13. For some of the sites, only leaf mines were recorded via observations. Larvae, puparia, adults and parasitoids were preserved in 75% ethanol. Most adults were dried using HMDS (hexamethyldisilazane). Photographs of the life histories and live specimens of both adults and immature stages were taken with digital cameras (Nikon Coolpix 4500, 8700, and Canon PowerShot G7). The final digital images were processed using Adobe Photoshop CS4. Type specimens are deposited in the following collections (acronyms used in the text are in parentheses): Canadian National Collection of Insects, Arachnids & Nematodes, Ottawa, ON, Canada (CNC); Museo de Zoología, Universidad de Costa Rica, San José, Costa Rica (MZUCR); Instituto Nacional de Biodiversidad, Santo Domingo de Heredia, Costa Rica (INBio); Lyman Entomological Museum, McGill University, Ste-Anne-de-Bellevue, QC, Canada (LEM); National Museum of Natural History, Smithsonian Institution, Washington, DC, USA (NMNH).

Specimens of immature stages are deposited in CNC and MZUCR, and parasitoid wasps in MZUCR.

**Table 1.** *Liriomyza* species occurrence at different site localities in Costa Rica. All from host plant *Bocconia frutescens* L., unless specified otherwise.

Sites	Province	Locality, Lat.-Long.	Elevation	Species	Imago ♂♀		Comments/ immature stages
1	Alajuela	Parque Nacional Volcán Arenal, 10°27'57"N, 084°45'18"W	600 m	*Liriomyza prompta*	0	0	1 puparium
	Alajuela	Parque Nacional Volcán Arenal, 10°27'54"N, 084°45'15"W	605 m	*Liriomyza prompta*	0	0	leaf mine
2	Alajuela	Reserva Biológica Manuel Alberto Brenes, open valley area, (Fig. 1) 10°13'43"N, 084°34'10"W	796 m	*Liriomyza mystica*	0	0	1 puparium
	Alajuela	Reserva Biológica Manuel Alberto Brenes, open valley area, (Fig. 1) 10°13'43"N, 084°34'10"W	796 m	*Liriomyza prompta*	2	2	larvae, puparia
	Alajuela	Reserva Biológica Manuel Alberto Brenes, 10°13'07"N, 084°35'49"W	850 m	*Liriomyza mystica*	0	0	1 puparium
	Alajuela	Reserva Biológica Manuel Alberto Brenes, 10°13'07"N, 084°35'49"W	850 m	*Liriomyza prompta*	0	1	oviposition, 1 puparium
3	Alajuela	Laguna de Hule, 10°18'14"N, 084°12'26"W	809 m	*Liriomyza mystica*	0	0	1 larva
	Alajuela	Laguna de Hule, 10°18'14"N, 084°12'26"W	809 m	*Liriomyza prompta*	0	0	1 puparium
4	Alajuela	Bajo del Toro, 10°08'27"N, 084°19'60"W	1380 m	*Liriomyza prompta*	0	0	leaf mines
5	Cartago	San Ramón de Tres Ríos, 09°56'20"N, 083°58'55"W	1500 m	*Liriomyza mystica*	1	5	larvae, puparia
	Cartago	San Ramón de Tres Ríos, 09°56'18"N, 083°58'38"W	1600 m	*Liriomyza mystica*	35	35	larvae, puparia
	Cartago	San Ramón de Tres Ríos, 09°56'18"N, 083°58'38"W	1600 m	*Liriomyza prompta*	13	7	larvae, puparia
	Cartago	San Ramón de Tres Ríos, 09°56'27"N, 083°58'16"W	1670 m	*Liriomyza mystica*	0	0	larvae
6	Cartago	Cervantes, 09°52'46"N, 083°49'07"W	1500 m	*Liriomyza prompta*	1	0	larvae, puparia
	Cartago	Cervantes, 09°52'46"N, 083°49'07"W	1500m	*Liriomyza mystica*	0	0	larvae
7	Cartago	Llano Grande, 09°54'41"N, 083°53'09"W	2100 m	*Liriomyza prompta*	0	0	leaf mines
8	Cartago	Chicuá, Irazú Volcano, 09°56'49"N, 083°52'00"W	2765 m	*Liriomyza mystica*	0	0	1 larva
9	Heredia	Santo Domingo de Heredia, 09°58'21"N, 084°05'29"W	1133 m	*Liriomyza prompta*	0	1	larvae, puparia, on *Bocconia arborea*
10	Heredia	Cinchona-Vara Blanca, (Fig. 2) 10°13'26"N, 084°09'47"W–10°11'02"N, 084°09'18"W	1200–1800m	*Liriomyza mystica*	0	0	larvae, puparia
	Heredia	Cinchona-Vara Blanca, (Fig. 2) 10°13'26"N, 084°09'47"W–10°11'02"N, 084°09'18"W	1200–1800m	*Liriomyza prompta*	0	0	larvae, puparia
11	Limón	Guácimo, 10°08'34"N, 083°42'27"W	480 m	*Liriomyza prompta*	0	0	leaf mines
12	Puntarenas	Bosque Eterno de los Niños, Bajo del Tigre, 10°30'31"N, 084°49'12"W	1130 m	*Liriomyza prompta*	0	2	larvae, puparia
13	Puntarenas	Estación Biológica Monteverde, 10°19'09"N, 084°48'32"W	1538 m	*Liriomyza prompta*	1	1	mating (Fig. 6)
	Puntarenas	Estación Biológica Monteverde, 10°19'09"N, 084°48'32"W	1538 m	*Liriomyza mystica*	1	1	mating, larvae and puparia
14	San José	San Pedro de Montes de Oca, 09°56'27"N, 084°02'36"W	1236 m	*Liriomyza prompta*	0	0	larvae, puparia
15	San José	San Isidro de Coronado, (Fig. 3) 09°58'18"N, 084°00'22"W	1420 m	*Liriomyza prompta*	6	12	larvae, puparia
	San José	San Isidro de Coronado, (Fig. 3) 09°58'18"N, 084°00'22"W	1420 m	*Liriomyza mystica*	0	1	larvae, puparia
16	San José	San Gerardo de Rivas area, 09°28'13"N, 083°35'07"W–09°27'51"N, 083°34'15"W	1457–2031 m	*Liriomyza prompta*	0	0	leaf mines
17	San José	Parque National Chirripó, forest fire area approx. 09°27'20"N, 083°31'59"W	ca. 2700 m	*Liriomyza mystica*	0	0	1 puparium, 1 larva

## Results

Both species of *Liriomyza* were present at most of the field sites, sometimes containing larvae of the two species on the same leaf. A total of 127 adult specimens representing two new species of *Liriomyza* were obtained from nine localities in Costa Rica (Table 1). Most of these specimens were reared from *Bocconia frutescens* except four specimens that were collected while mating under leaves of *Bocconia frutescens*, and two reared from *Bocconia arborea* (Table 1). No adult specimens were successfully reared from some of the sites, but species identification was still possible with larvae and/or puparia obtained. Species description and details on biology follow.

### Liriomyza mystica

Boucher & Nishida sp. n.

http://zoobank.org/48883C22-4ED7-438D-B6F4-1A290B51763F

http://species-id.net/wiki/Liriomyza_mystica

Figs 4 ,8 –27 ,48 ,50

#### Type material.

Holotype ♂: COSTA RICA: Cartago: San Ramón de Tres Ríos, 1600 m, (09°56'18"N, 083°58'38"W), ex. *Bocconia frutescens*, larva exited 5–9.vii.2010; adult emerged 21–26.vii.2010, Kenji Nishida (LEM).

Paratype: same data as holotype (9 ♂; 8 ♀: LEM); same except larva exited: 25–28.vi.2010, adult emerged: 20–22.vii.2010 (14 ♂; 15 ♀: INBio); same except larva exited 29.vi–4.vii.2010, adult emerged 15–24.vii.2010 (8 ♂; 7 ♀: NMNH); same except adult emerged 21–25.vii.2010 (4 ♂; 4 ♀: CNC); same except along main road, (09°56'20"N, 083°58'55"W), 1500 m, collected 19.xii.2008, emerged 16–20.i.2009, K. Nishida & T. Johnson (1 ♂; 5 ♀: MZUCR). San José: San Isidro de Coronado, Centro. 1420 m, (09°53'18"N, 084°00'22"W), ex. *Bocconia frutescens*, adult emerged 17.vi.2010, Kenji Nishida (1 ♀: LEM). Puntarenas: Monteverde. Estación Biológica Monteverde. 1538 m, (10°19'09"N, 084°48'32"W), mating on *Bocconia* leaf. 18.vii.2010, Kenji Nishida (1 ♂; 1 ♀: MZUCR).

#### Diagnosis.

This species can be distinguished from other Neotropical species of *Liriomyza* by its completely yellow head and anepisternum, mesonotum almost completely brown to margin of scutellum, usually 2 + 1 dc, legs completely yellow, calypter brown on apical half with margin and fringe brown, and by the shape of the male genitalia and the shape of the anterior and posterior larval spiracles.

#### Description.

**Figures 8–11.**
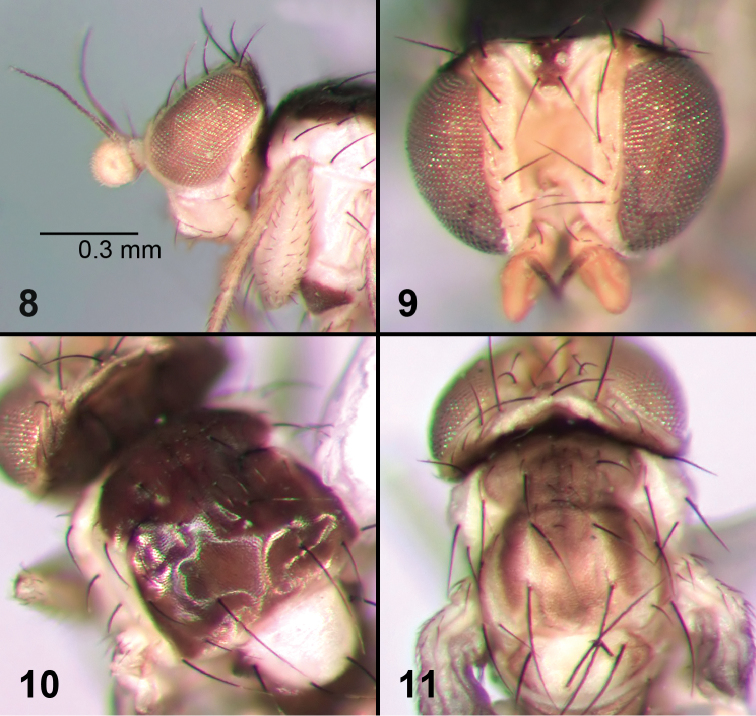
External morphology of adult *Liriomyza mystica*. **8** Head, lateral **9** Head, dorso-frontal **10** Thorax, dorsal **11** Thorax, dorsal (teneral specimen).

Frons width 0.25 mm; ratio of frons width to eye width 2.3; orbit 0.23 times width of frons at midpoint; frons slightly projecting above or in front of eye in profile (Fig. 8), forming a distinct ring (cheek) below eye; 2 reclinate *ors* and 2 inclinate *ori* (Fig. 9) (lower ori sometimes reduced or missing on one side); orbital setulae reclinate, varying in number from about 4–8; first flagellomere rounded, not enlarged in males, with slight apical pubescence; arista 0.30–0.40 mm, with short but dense pubescence; gena deep, slightly extended at rear (Fig. 8); gena height at midpoint: 0.44 times maximum eye height. Eye oblique, bare. Normally 2+1 dc (except 4 specimens with 2+0 dc and 3 specimens with 3+1 dc); acrostichals in about 4 irregular rows; prescutellar acrostichal bristles absent; 2 notopleural bristles; 1 strong postpronotal bristle with 1 or 2 small setulae; anepisternum with 1 strong bristle on posterior margin at midpoint, sometimes with a few extra setulae; katepisternum with one strong bristle on posterodorsal corner, on yellow ground. Fore and mid-tibia without lateral bristle. Wing length 1.50–1.95 mm in male and 1.85–2.20 mm in females; M_1+2_ ending at wing tip; costa extending to M_1+2_; last section of CuA_1_: 1.5–1.9 times length of penultimate. Cross-vein r-m located at midpoint of cell dm. Stridulatory mechanism apparently absent.

**Colour.** Head (including frons, orbit, face, antenna, palp) entirely bright yellow. Hind margin of eye black for a small section beyond vte; both vt on yellow ground. Occiput black. Eye sometimes with a slight bluish or greenish reflection (not as pronounced as in *Liriomyza prompta*, Fig. 29); mesonotum almost completely dark brown except for narrow yellow margin posteriorly (Fig. 10), prescutellar area and intra-alar area sometimes slightly paler brown resulting in a weakly defined banded pattern on thorax, most visible in teneral specimen (Fig. 11); scutellum completely yellow with small brown patches laterally. Basal scutellar bristles on brown ground (but at the limit of yellow). Postpronotum, notopleuron and anepisternum completely yellow (at most with a very small pale brown patch on one or two of the sclerites). Katepisternum mostly brown except for upper margin yellow. Calypter brown on apical half, margin and fringe also brown; halter completely white. Legs completely yellow. Abdominal tergites pale brown.

**Figures 12–15.**
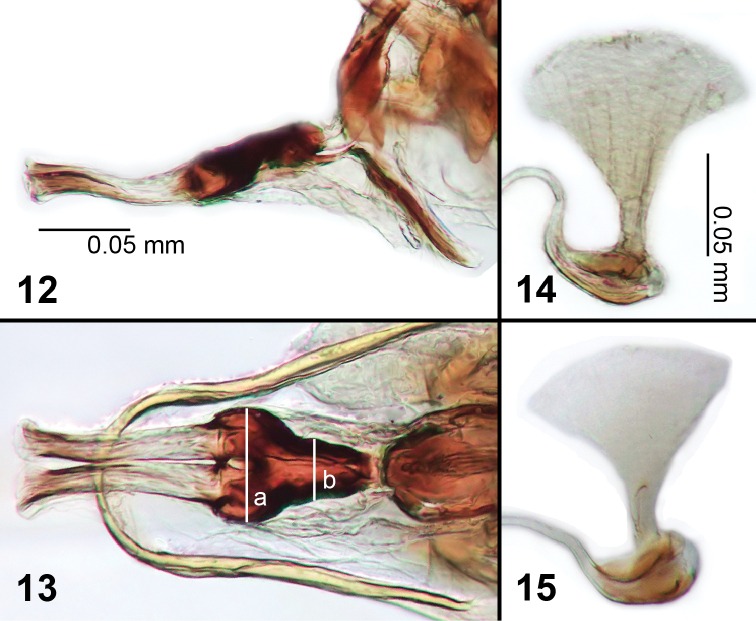
Male genitalia of *Liriomyza mystica*. **12** Phallus, lateral **13** Phallus, ventral (see text for lines ‘a’ and ‘b’) **14, 15** Ejaculatory apodeme.

**Male genitalia.** Distiphallus in the form of two narrow tubules, slightly diverging apically in ventral view (Fig. 13). Mesophallus widest apical section (Fig. 13a), about 1.5–2 times larger than basal narrower tubular section (Fig. 13b). Mesophallus in lateral view with small indent at midpoint (Fig. 12). Surstylus absent. Epandrium without chitinized margin and without spines. Ejaculatory apodeme (Figs 14, 15) weakly sclerotized, symmetrical or sometimes asymmetrical with blade more expanded on one side.

**Figures 16–19.**
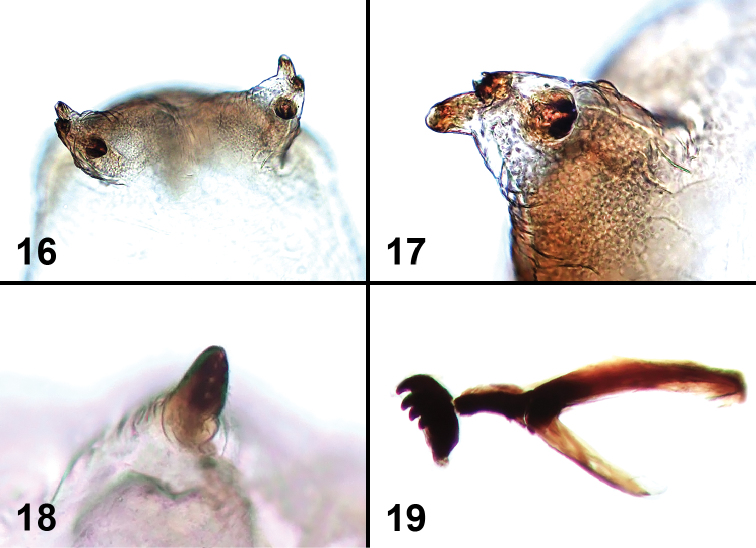
Larval characters of *Liriomyza mystica*. **16** Posterior spiracles **17** Posterior spiracle (close-up) **18** Anterior spiracle (note angle of view is different from Fig. **38**) **19** Cephalopharyngeal skeleton.

**Early stages.** Larval length (at maturity): 3.2–4.0 mm, slightly larger than *Liriomyza prompta* larva. White to creamy white with an internal orange spot at head (live specimens, Figs 24, 25, 27). Anterior spiracles about 0.13–0.23 mm distance from each other; fan-shaped and each with 5 small openings in a single row (Fig. 18). Posterior spiracles divided into 3 subequal projecting bulbs (Figs 16, 17). Cephalopharyngeal skeleton with wide arms (Fig. 19), Each mandible with 2 large teeth. Puparium pale brown to transparent (Fig. 48).

**Figures 20–27.**
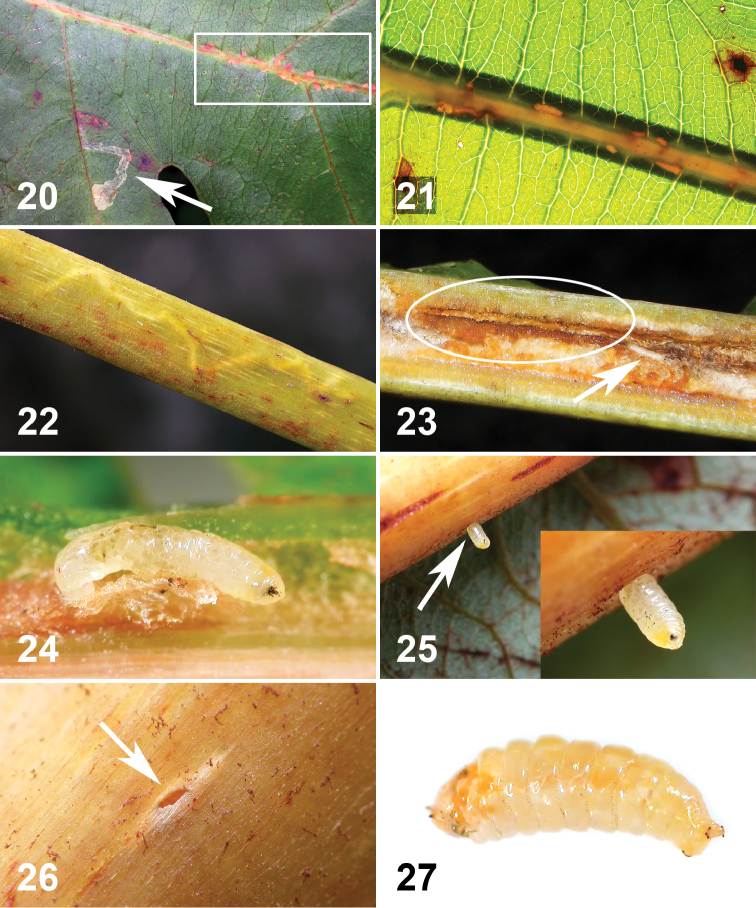
Life history of *Liriomyza mystica* larvae on *Bocconia frutescens*. **20–22** External evidence caused by internal larval feeding on vein and petiole **20** Brown to reddish brown spots (ca. 1–2 mm long) on upperside along primary vein, marked by rectangular line. Arrow indicates *Liriomyza prompta* mine **21** Pale brown linear spots along the primary vein seen through strong sunlight from the back. Note that lower part of vein (underside) is thicker and shown as shadow **22** Mine in pale colour zigzag, approximately 30 mm long **23** Longitudinally opened primary vein with linear mine (circle) and late instar larva (arrow) **24** Late instar larva in situ, ventral view. Cephalopharyngeal skeleton on right. Notice orange spot at head **25** Mature larva exiting from underside of vein (arrow). Close-up view, lower right. Notice orange spot at head **26** Exit hole (ca. 1 mm wide) on underside of primary vein **27** Mature larva in pre-puparial stage. Posterior on right.

#### Host plant.

*Bocconia frutescens* L. (Papaveraceae).

#### Biology.

The larvae feed on spongy parenchyma and other tissues of primary veins and petioles of large, relatively old, mature leaves. Most of the larvae were in leaves of >30 cm long, with >10 mm petiole width and >10 mm thickness (n=120). The larvae were more frequently found mining in the thicker part of the primary vein including the petiole (i.e. less frequently near the leaf apex). One larva was found mining inside of a 2.2 mm width primary vein near the leaf apex. A few larvae were mining thick secondary veins. The mining appears to occur longitudinally, mostly near the upper leaf surface; the larvae left some brown to reddish brown scars along the leaf blade where the vein and blade join (Figs 20, 21, 23). These scars were more easily seen with a strong transmitted light (Fig. 21). The mines (internal tunnels) can also be distinguished by narrow pale lines (Fig. 22). When infested veins were longitudinally dissected, usually one to a few white *Liriomyza prompta* larvae were observed mining singly and scattered (n=12 leaves) (Figs 23, 24). Some solitary parasitoid wasp pupae were also found among the spongy parenchyma (Fig. 50). The mature fly larvae exited from either the upper side or underside of the veins (n=12 holes) (Fig. 25), each larva making a small oval-shaped hole of 1.1–1.3 mm wide (n=12) (Fig. 26). The tissue around the old exit holes was brown to reddish brown (n=5). The newly emerged larvae wiggled around in rearing plastic bags/cases for a couple of hours to a few hours before settling (Fig. 27) and starting to form a puparium. The larvae readily pupated on the plastic surfaces. In general, the puparia (Fig. 48) were more translucent (translucent pale brown) than those of *Liriomyza prompta* (translucent brown to dark brown) and the pupa inside was visible. Duration of the larval stage was not recorded. The larvae that exited from veins pupated between 26 to 29.vi.2010 and the adults emerged between 20 to 22.vii.2010, i.e. the pupal stage lasted approximately 25 days (n=29). A mating pair was observed on the underside of a leaf around 7:00 am (site 13). No oviposition behavior was observed for this species.

#### Parasitoids.

Two species of Pteromalidae: Pteromalinae: sp. 01 from sites 5, 10, 15, parasitizing late instar larva, pupating inside the leaf vein (Fig. 50); Pteromalinae sp. 02 from sites 5, 10, 13, parasitizing larva and pupating inside the host puparium; one species of Braconidae: Opiinae: *Opius* sp. from site 10, parasitizing larva and pupating inside the host puparium.

#### Comments.

*Liriomyza mystica* is most similar to *Liriomyza prompta* described below and to the Neotropical species *Liriomyza commelinae* (Frost) and *Liriomyza robustae* Spencer, especially in the form of the phallus with paired tubules. But these two latter species differ from *Liriomyza mystica* in a number of characters, including their host plants (both known from plants in the family Commelinaceae); mesonotum with a distinctive black and yellow pattern; surstylus with a distinct spine; third antennal segment enlarged in males; shape of both anterior and posterior spiracles and pupation occurring inside the mine (Silva and Oliveira 1952, Spencer 1984, Valladares 1984). The anterior and posterior spiracles of *Liriomyza mystica* are most similar to those of *Liriomyza caesalpiniae* Valladares reared from a Caesalpiniaceae, *Caesalpinia gilliesii* Benth. (Valladares 1984: figs 14, 15).

Most adult specimens of *Liriomyza mystica* were reared from site 5, but it was also found at other sites, up to an elevation of 2765 m (Table 1). Considering that *Liriomyza mystica* larvae feed inside primary veins and petiole of large, mature leaves, it made it difficult to establish *Bocconia arborea* as possible host due to the problems in studying large trees with large leaves. In sapling trees of ca. 1 m tall (n=2) at Santo Domingo de Heredia (site 9), no larvae or evidence of feeding was observed.

#### Etymology.

The species name is derived from the Latin *mysticus* (secret, mystic), referring to the hidden and inconspicuous leaf mines in primary vein and petiole.

### Liriomyza prompta

Boucher & Nishida sp. n.

http://zoobank.org/5933AA4E-ACDE-4A2F-8F66-4CD9AA55F1C6

http://species-id.net/wiki/Liriomyza_prompta

Figs 5–7 ,20 ,28 –47 ,49 ,51

#### Type material.

Holotype ♂: COSTA RICA: Cartago: San Ramón de Tres Ríos, 1600 m (09°56'18"N, 083°58'38"W), ex. leaf mine *Bocconia frutescens*, larva exited 5–9.vii.2010, adult emerged 21–26.vii.2010, Kenji Nishida (LEM).

**Paratype.** same data as holotype (2 ♀: LEM); same except: ex. leaf mine *Bocconia frutescens*, emerged 1.vi.2010, Kenji Nishida (1 ♀: LEM); same except larva exited 29.vi.2010, adult emerged 20.vii.2010 (1 ♀: LEM); same except larva exited 29.vi–4.vii.2010, adult emerged 15–24.vii.2010 (7 ♂; 1♀: LEM); same except larva exited 25–28.vi.2010, adult emerged 20–22.vii.2010 (1 ♀: NMNH); same except larva exited 29.vi–4.vii.2010, adult emerged 21–25.vii.2010 (5 ♂; 1 ♀: NMNH); Cartago: Cervantes, 1500 m (09°52'46"N, 083°49'07"W), ex. *Bocconia frutescens*, emerged 11.vi.2010, Kenji Nishida (1 ♂: CNC); San José Province: San Isidro de Coronado, Centro 1420 m (09°58'18"N, 084°00'22"W), ex. *Bocconia frutescens*, adult emerged 11.vi.2010, Kenji Nishida (1 ♀: MZUCR); same except puparia formed 10–12.v.2010, adult emerged 12.vi.2010, Kenji Nishida (1 ♀: MZUCR); same except emerged 10.vi.2010 (1 ♂: INBio); same except pupation 6.vi.2010, emerged 26.vi.2010, Kenji Nishida (1 ♀: INBio); same except collected 15.v.2009, emerged 8.vi.2009, K. Nishida (4 ♂; 6 ♀: MZUCR), same except (1 ♂; 3 ♀: CNC); Puntarenas Prov., Monteverde, Estación Biológica, 1538 m (10°19'09"N, 084°48'32"W), mating on *Bocconia frutescens* leaf, 30.v.2009, Kenji Nishida (1♂; 1♀: INBio).

#### Diagnosis.

This species can be distinguished from other Neotropical species of *Liriomyza* by its completely yellow head and anepisternum, mesonotum almost completely brown to margin of scutellum, usually 3 + 1 dc, legs completely yellow, calypter brown on apical half with margin and fringe brown, and by the shape of the male genitalia and the shape of the anterior and posterior larval spiracles.

#### Description.

**Figures 28–31.**
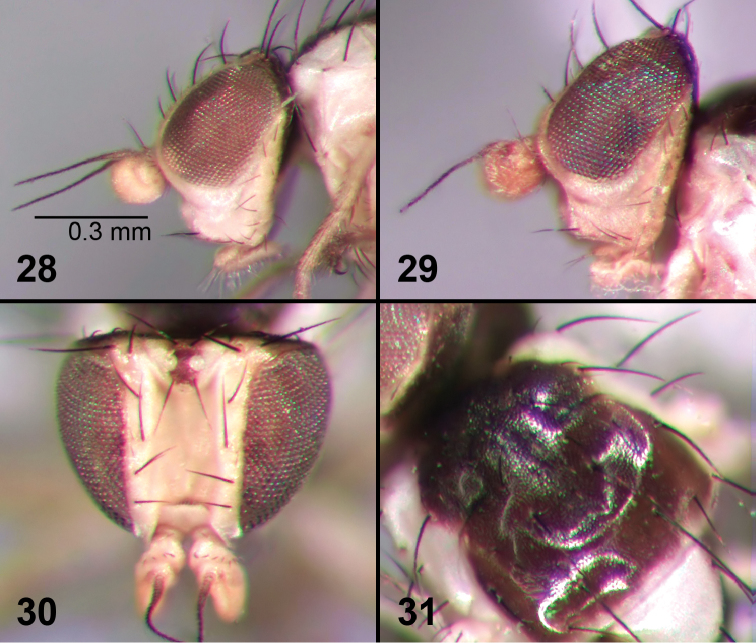
External morphology of adult *Liriomyza prompta*. **28** Head, lateral **29** Head, lateral (variation of eye colour) **30** Head, dorsal **31** Thorax, dorsal.

As in *Liriomyza mystica* Boucher and Nishida (described above) except as follows: arista shorter (Figs 28, 29), 0.23–0.3 mm; normally 3+1 dc (except one male specimen with 2+1 dc); generally smaller: wing length 1.4–1.7 mm in males and 1.6–2.2 mm in females; eye often with a more extensive bluish reflection (Fig. 29).

**Male genitalia.** Similar to *Liriomyza mystica*, but tubules of distiphallus more sclerotized, slightly wider, parallel sided (not diverging in ventral view). Mesophallus widest apical section (Fig. 33a), more than twice as large as narrower basal tubular section (Fig. 33b). Mesophallus in lateral view with a prominent curve near midpoint. Surstylus absent. Ejaculatory apodeme (Figs 34, 35) slightly more sclerotized than in *Liriomyza mystica*, and with blade slightly less expanded.

**Figures 32–35.**
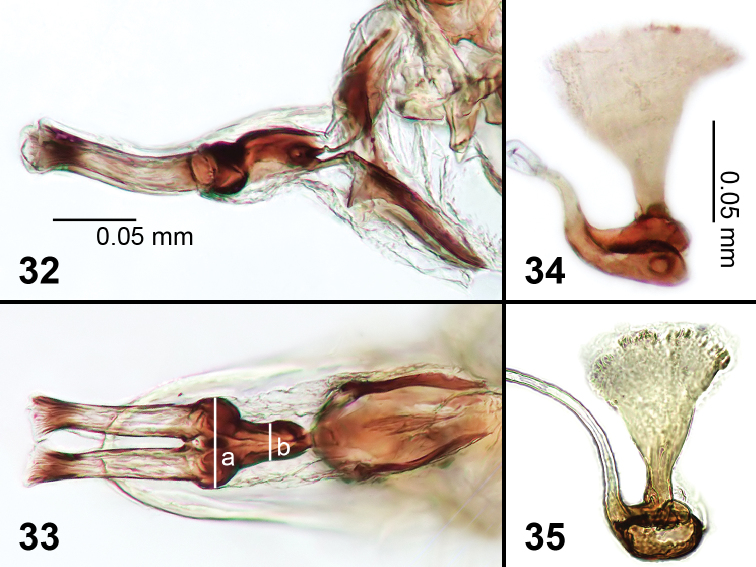
Male genitalia of *Liriomyza prompta*. **32** Phallus, lateral **33** Phallus, ventral (see text for lines ‘a’ and ‘b’) **34, 35** Ejaculatory apodeme.

**Figures 36–39.**
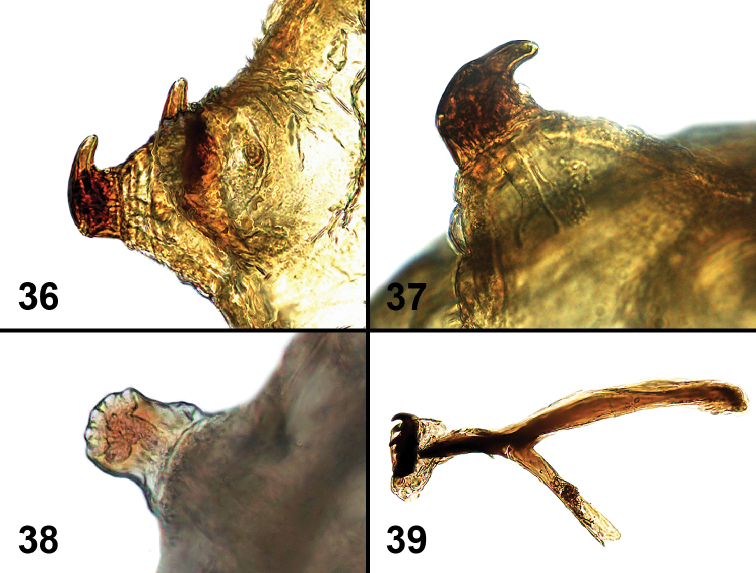
Larval characters of *Liriomyza prompta*. **36** Posterior spiracles **37** Posterior spiracle (close-up) **38** Anterior spiracle (note angle of view is different from Fig. **18**) **39** Cephalopharyngeal skeleton.

**Early stages.** Larval length: 1.95–2.70 mm, slightly smaller than *Liriomyza mystica* larva. White to creamy white with an internal orange spot at head (Fig. 47). Anterior spiracles about 0.1 mm distance from each other. Similar to *Liriomyza mystica*: fan-shaped with 5 small openings. Posterior spiracles with apparently 3 bulbs, but two very small and 1 much longer, curving toward anal segment (Figs 36, 37). Cephalopharyngeal skeleton (Fig. 39) more elongated with side arms narrower than in *Liriomyza mystica*. Puparium translucent brown to dark brown (Fig. 49).

**Figures 40–47.**
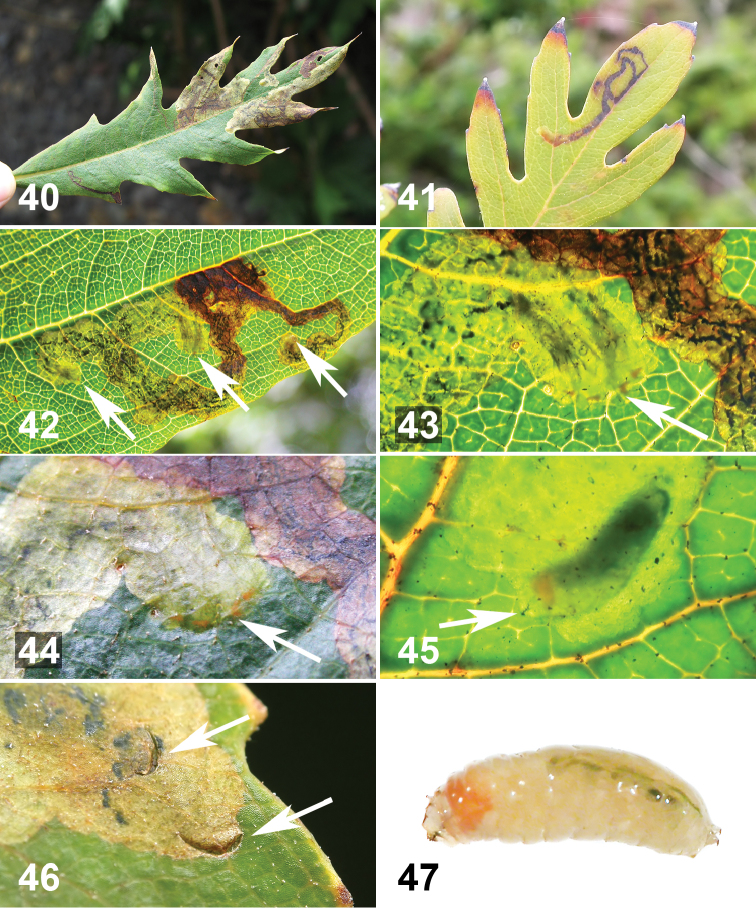
Life history of *Liriomyza prompta* larva on *Bocconia frutescens* leaves **40** Leaf mines on upperside of mature, but relatively small leaf. Note that there are both single (narrow linear) and gregarious (blotch) mines **41** Active single larval mine at site 17. Note that early part of the mine becomes brown **42** Close-up of active mine with 7 larvae, seen with transmitted light (arrows indicate groups of late instar larvae): 2, 4, and 1 larvae from left to right). Early part of mine brown. Frass in dark green to black linear dots **43** Close-up of middle arrow area of Figure **42**, arrow indicates four actively mining larvae. Notice four orange spots **44** Same as Figure **43**, but without transmitted light **45** Close-up of actively mining late instar larva. Arrow indicates cephalopharyngeal skeleton **46** Exit holes (arrows), approximately 1.2 mm wide, near or at end of mine **47** Mature larva recently exited from mine. Posterior on right.

**Figures 48–51.**
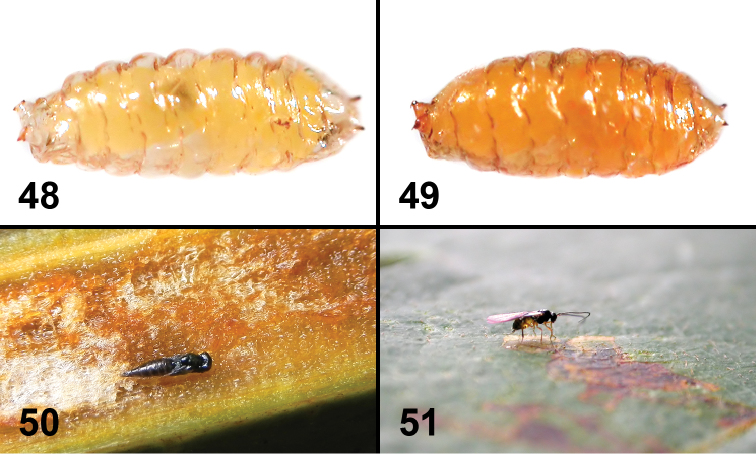
Life history of two new species of *Liriomyza*. **48** Puparium of *Liriomyza mystica*, in situ **49** Puparium of *Liriomyza prompta*, in situ **50** Pupa of Pteromaline parasitoid wasp (sp. 01) inside *Bocconia frutescens* leaf vein parenchyma **51** Braconid parasitoid wasp, most likely *Opius* sp., attempting to oviposit in mature *Liriomyza prompta* larva at site 13.

#### Host plants.

*Bocconia frutescens* L. and *Bocconia arborea* S. Watson (Papaveraceae)

#### Biology.

At site 15 in late October 2012 a few males were perched on small young leaves at the apical shoot of a 2 m host tree in the morning between 6:00 and 7:00 am. The males were either sitting or flying and perching on apical young leaves. A mating couple was observed on the underside of host leaf at 7:00 am (site 13, Fig. 6). Oviposition was observed a few times by two females at site 2. At 3:00 pm (17.vi.2011), overcast with slight drizzle, the two females were walking on the upper side of leaves of a *Bocconia frutescens* sapling, ca. 50 cm tall. The females either oviposited in the leaf tissue at the edge of the leaf margin (n=2) or along narrow leaf veins on the upper side of the leaf blade (n=3) (Fig. 7). It was difficult to locate the eggs because of their small size and translucent colour (n=1). After oviposition, the leaf was collected and a small larva was found on 28.vi.2011. Also at site 5, three leaves were randomly collected on 24.vi.2010, and newly started mines were observed on 2–3.vii.2010 (n=6 mines). These observations suggest that the duration of the egg stage is approximately 10 days. The larvae mine mesophyll of the leaf blade either singly or gregariously (up to 7 larvae) (n=70 mines) (Figs 40–45). Mines were found on very small mature leaves of ca. 1.5 cm (n=5, at site 13) to 90 cm long (n=10, at site 10). The mature mines with a single larva were narrow and more or less linear (Figs 20, 40, 41), and mines with multiple larvae were blotch-shaped (Figs 40, 42). These mines were conspicuous on the upper side of leaves. With regard to location of mines on leaves, no patterns were noticed; mines appear to occur on any part of the leaf blade — some mines were found near the primary vein, leaf apex, or anywhere in between (uncounted). Presence of the larva in the mine can be recognized by the orange spot of the larval anterior end (Figs 43–45, 47). The early part of the mine becomes brown and the frass is seen in dark green to black linear dots (Figs 42–44). The mature larvae each made an elliptical exit hole 1.1–1.3 mm wide on the upper side of the leaves, (n=7 holes) (Fig. 46). Under rearing conditions, from the time of recognizing very early mines, the larvae completed mining within 3 to 4 days, and on the 5th day the puparia were usually formed on the plastic surface of rearing bags or containers. Young pupae were observed within a day or two after forming of the puparium (n=16). The puparia (Fig. 48) were less translucent than those of *Liriomyza mystica*. The pupa becomes dark 2–3 days prior to adult emergence. A cohort of larvae from site 5 which pupated on 20–23.xii.2008 emerged as adults on 16–20.i.2009 (n=6, 1 ♂, 5 ♀), other data as follows: pupation 28.vi.2011, adult emergence 24.vii.2011 (a cohort, n=4, 2 ♂, 2 ♀, site 2); pupation 12–13.ii.2012, emergence 8.ii.2012 (n=2, site 12); pupation 20.iv.2012, emergence 14.v.2012 (n=2, site 15); i.e. the pupal stage lasted nearly a month under the rearing conditions. A small population of *Bocconia arborea* was found at site 9; however, it was only possible to access two saplings, which were about 1 meter tall and infested by *Liriomyza prompta* larvae (mostly old mines). Also at sites 7, 9 and 15, populations of weedy Papaveraceae, *Argemone mexicana* L. were found in close proximity to *Bocconia frutescens* and *Bocconia arborea*; however, no leaf mines of *Liriomyza prompta* were observed.

#### Parasitoids.

Three species of Eulophidae: Entedoninae: sp. 01 and sp. 02 from sites 5, 6, 13, and 15, Entedoninae sp. 03 from site 15, all these were parasitizing larva and pupating inside the host puparium; Eulophinae: sp. 01 from sites 13 and 15, parasitizing late instar larva, pupating inside the host leaf mine; and two species of Pteromalinae as mentioned in *Liriomyza mystica*, sp. 01 from site 5 and 15, and sp. 02 from site 13 and 15. A species of Braconidae, probably an Opiinae: *Opius* sp. was observed parasitizing a mature *Liriomyza prompta* larva at site 13 (Fig. 51).

#### Comments.

An important character differentiating larvae of this species from *Liriomyza mystica* described above, is that the posterior spiracles each have an elongated, somewhat hook-like bulb. These posterior spiracles are similar to those found in the Neotropical species *Liriomyza commelinae* and *Liriomyza robustae*, but the uniformly coloured mesothorax, absence of surstyli, and unforked anterior spiracles, differentiate this new species from these other Neotropical species. This species appears to be more common than *Liriomyza mystica* with a wider elevation range (Table 1).

#### Etymology.

The species name is derived from the Latin *promptus* (visible, apparent), referring to their conspicuous and common leaf mines.

## Supplementary Material

XML Treatment for Liriomyza mystica

XML Treatment for Liriomyza prompta

## References

[B1] Benavent-Corai J Martinez M Jiménez-Peydró R (2005) Catalogue of the hosts-plants of the world Agromyzidae (Diptera). Bollettino Di Zoologia Agraria E Di Bachicoltura. Serie II Vol 37 Supplementum.

[B2] Benitez DM Saulibio D (2007) *Bocconia frutescens* distribution on the island of Hawai`i. Technical Report 144 University of Hawai`i at Manoa, Pacific Cooperative Studies Unit.

[B3] Boucher S (2010) Family Agromyzidae (leaf-mining flies). In: Brown BV Borkent A Cumming JM Wood DM Woodley NE Zumbado M (Eds) Manual of Central American Diptera. Volume 2 National Research Council Press, Ottawa, 1057–1071.

[B4] Hammel BE Grayum MH Herrera C Zamora N (Eds) (2007) Manual de Plantas de Costa Rica, Volumen VI, Dicotiledoneas (Haloragaceae-Phytolaccaceae). Monographs in Systematic Botany from the Missouri Botanical Garden 111: 1-933. [in Spanish]

[B5] INBio (1997–2006) Instituto Nacional de Biodiversidad Atta. http://www.inbio.ac.cr/ [accessed 2 September 2008]

[B6] Martinez M Etienne J (2002) Liste systématique et biogéographique des Agromyzidae (Diptera) de la région néotropicale. Bolletino di Zoologia agraria e di Bachicoltura, Serie II, 34: 25-52.

[B7] Parrella MP (1987) Biology of *Liriomyza*. Annual Review of Entomology 32: 201-224. doi: 10.1146/annurev.en.32.010187.001221

[B8] Silva GA Oliveira SJ (1952) Sobre um «Agromyzidae» (Diptera) cujas larvas minam Fohas de Trapoeiraba (Commelinaceae). Revista Brasileira de biologia, 293–299.

[B9] Spencer KA (1973) Agromyzidae of economic importance. Series entomologica 9 W. Junk B.V., The Hague, 418 pp. doi: 10.1007/978-94-017-0683-4

[B10] Spencer KA (1984) The Agromyzidae (Diptera) of Colombia, including a new species attacking potato in Bolivia. Revista Colombiana de Entomología 10(1–2): 3-33.

[B11] Spencer KA (1990) Host specialization in the World Agromyzidae (Diptera). Series Entomologica 45 Kluwer Academic Publishers, Dordrecht, 444 pp. doi: 10.1007/978-94-009-1874-0

[B12] Valladares G (1984) Sobre el género *Liriomyza* Mik, 1894 (Diptera, Agromyzidae) en la República Argentina. Revista de la Sociedad Entomológica Argentina 43: 13-36.

[B13] Wagner WL Herbst DR Sohmer SH (1999) Manual of the Flowering Plants of Hawai`i. 2 vols. Bishop Museum Special Publication 83, University of Hawai`i and Bishop Museum Press, Honolulu, HI.

[B14] Wester L (1992) Origin and distribution of adventive alien flowering plants in Hawai`i. In: Stone CP Smith CW Tunison JT (Eds) Alien Plant Invasions in Native Ecosystems of Hawai`i. University of Hawai`i Press, Honolulu, HI.

